# Desk-based workers’ perspectives on using sit-stand workstations: a qualitative analysis of the Stand@Work study

**DOI:** 10.1186/1471-2458-14-752

**Published:** 2014-07-25

**Authors:** Josephine Y Chau, Michelle Daley, Anu Srinivasan, Scott Dunn, Adrian E Bauman, Hidde P van der Ploeg

**Affiliations:** Prevention Research Collaboration, Sydney School of Public Health, University of Sydney, Medical Foundation Building (K25), Camperdown, NSW 2006 Australia; Heart Foundation New South Wales, 3/80 William Street, 2011 Sydney, NSW Australia; Department of Public and Occupational Health, EMGO Institute for Health and Care Research, VU University Medical Center, van der Boechorststraat 7, 1081 BT Amsterdam, The Netherlands

## Abstract

**Background:**

Prolonged sitting time has been identified as a health risk factor. Sit-stand workstations allow desk workers to alternate between sitting and standing throughout the working day, but not much is known about their acceptability and feasibility. Hence, the aim of this study was to qualitatively evaluate the acceptability, feasibility and perceptions of using sit-stand workstations in a group of desk-based office workers.

**Methods:**

This article describes the qualitative evaluation of the randomized controlled cross-over Stand@Work pilot trial. Participants were adult employees recruited from a non-government health agency in Sydney, Australia. The intervention involved using an Ergotron Workfit S sit-stand workstation for four weeks. After the four week intervention, participants shared their perceptions and experiences of using the sit-stand workstation in focus group interviews with 4–5 participants. Topics covered in the focus groups included patterns of workstation use, barriers and facilitators to standing while working, effects on work performance, physical impacts, and feasibility in the office. Focus group field notes and transcripts were analysed in an iterative process during and after the data collection period to identify the main concepts and themes.

**Results:**

During nine 45-min focus groups, a total of 42 participants were interviewed. Participants were largely intrinsically motivated to try the sit-stand workstation, mostly because of curiosity to try something new, interest in potential health benefits, and the relevance to the participant’s own and organisation’s work. Most participants used the sit-stand workstation and three common usage patterns were identified: task-based routine, time-based routine, and no particular routine. Common barriers to sit-stand workstation use were working in an open plan office, and issues with sit-stand workstation design. Common facilitators of sit-stand workstation use were a supportive work environment conducive to standing, perceived physical health benefits, and perceived work benefits. When prompted, most participants indicated they were interested in using a sit-stand workstation in the future.

**Conclusions:**

The use of a sit-stand workstation in this group of desk-based office workers was generally perceived as acceptable and feasible. Future studies are needed to explore this in different desk-based work populations and settings.

## Background

Recently, sedentary behaviour has been emerging as a potential health risk behaviour for premature mortality and chronic health conditions such as cardiovascular disease and diabetes mellitus, even when physical activity is taken into account [1–5]. Sedentary behaviour is defined as activities that are done sitting or reclining and cost ≤1.5 times the basal metabolic rate [6].

Energy expenditure at work has decreased, with workers becoming more sedentary and less active over the past 50 years [7], and this trend has been projected to continue to 2030 [8]. Among Australian workers, 42% and 47% of men and women, respectively, characterise their jobs as involving ‘mostly sitting’, and those working full-time in such jobs sit for 6.3 h/day at work, on average [9]. In light of the increasingly sedentary nature of modern work and the high levels of occupational sitting time, it is important to address sedentary behaviour as part of workplace health promotion.

The workplace has been highlighted as an important setting for health promotion globally and nationally [10]. Workers in desk-based occupations are considered a key target group for workplace sitting reduction strategies [11, 12]. The current literature indicates that physical activity workplace interventions are not effective for specifically addressing workers’ sedentary behaviour and also highlights a paucity of workplace interventions that focus specifically on reducing workers’ sitting time [13].

Modifying the workplace environment by installing sit-stand workstations is one potential approach for reducing prolonged sitting among desk-based workers during working hours. Especially, as standing time has recently been positively associated with lower all-cause and cardiovascular disease mortality [14]. Much research on sit-stand workstations has been from the perspective of occupational ergonomics, related to musculoskeletal health and physical discomfort [15], and not to the prevention of cardiovascular and metabolic diseases. While research has begun to quantitatively examine the effectiveness of sit-stand workstations for reducing sitting and increasing standing during working hours as a strategy for chronic disease prevention [16–19], to our knowledge, only one study thus far has collected detailed qualitative data to examine user experiences and perspectives related to sit-stand workstations [17]. Hence, the aim of this study was to qualitatively evaluate the acceptability, feasibility and perceptions of using sit-stand workstations in a group of desk-based office workers in Sydney, Australia.

## Methods

The study was approved by the University of Sydney Human Research Ethics Committee (No. 08-2011/14067) and all participants gave written informed consent. The study is registered with the Australian New Zealand Clinical Trials Registry (No. ACTRN 12612000072819). This qualitative evaluation is one component of the Stand@Work study. Details about the Stand@Work study and its quantitative evaluation are provided elsewhere [20]. We report this qualitative study following the RATS qualitative research review guidelines (http://www.biomedcentral.com/authors/rats).

### Participants and design

**Figure 1:**
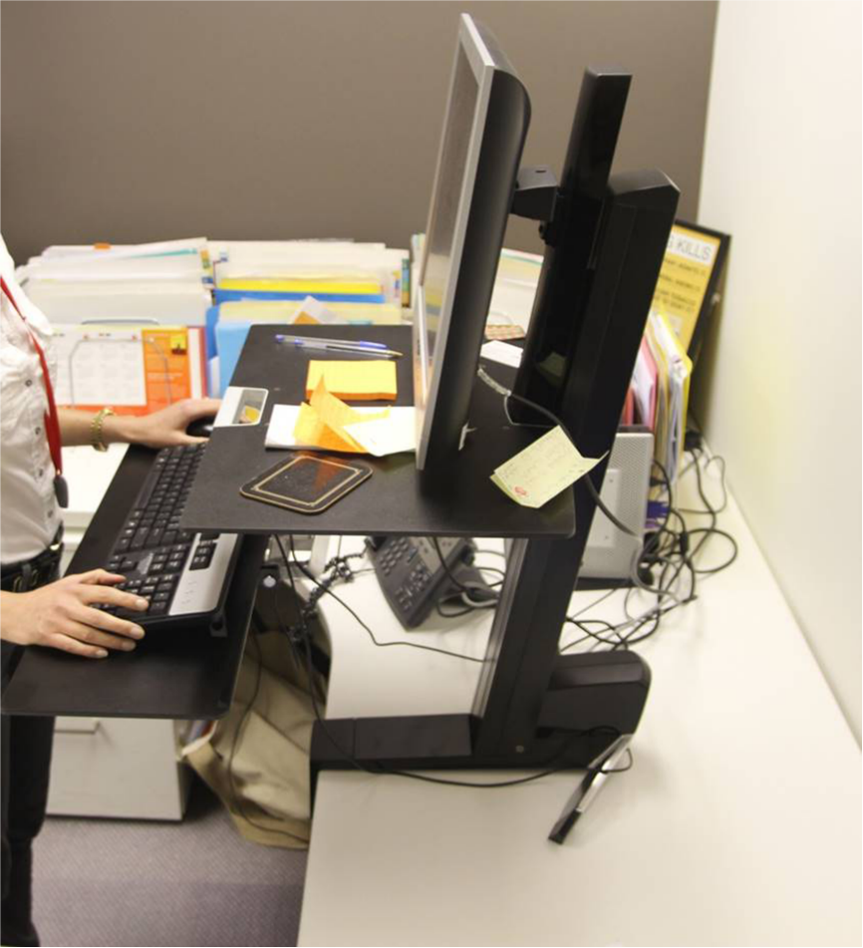
**Ergotron Workfit S sit-stand workstation.**

Participants were employees recruited from a non-government health agency in Sydney, Australia. Inclusion criteria included: aged at least 18 years old, working at least three days per week, and with sufficient English language proficiency to complete study tasks. The project was advertised to staff as part of their workplace wellness program and interested employees contacted the research team, who provided additional study information and an expression of interest form. Eligible staff members who returned an expression of interest form were randomly drawn from a ballot by a researcher in the presence of potential participants and other researchers, and were included in the study after providing written informed consent. The first four participants drawn from the ballot were allocated to the intervention group to use a sit-stand workstation for four weeks, the next four participants drawn from the ballot served as the control group. The remaining participants were assigned to the waitlist control condition and were placed on the waiting list in seven groups (4–5 people per group). After the initial four weeks, the previous control group (study group 2) received the intervention with the next group from the ballot draw serving as their controls (study group 3). This was repeated until all nine groups had received the intervention. This study design was used to maximise the evaluation sample size taking into account the five available sit-stand workstations. The intervention involved using an Ergotron model Workfit S sit-stand workstation (Figure 1) for four weeks. Users were provided with a demonstration and instructions on the use of the device, but there was no accompanying behavioural intervention. The study design and recruitment is described in more detail elsewhere [20]. The current manuscript adhered to the RATS guidelines for reporting qualitative studies.

### Procedures

After trialling the sit-stand workstation for four weeks, intervention participants attended a focus group facilitated by two members of the research team who were located at their workplace. One researcher facilitated each focus group discussion (MD), while the other took notes (AS). All focus groups were digitally recorded and transcribed verbatim for the purpose of analysis. We conducted focus groups rather than individual interviews due to time and resource constraints.

Each focus group discussion involved four to five participants and ran for approximately 45 minutes. Using a pre-prepared question guide (Table 1), participants were invited to share their views on a range of issues related to their experience with using the sit-stand workstation (e.g., patterns of workstation use, barriers and facilitators to standing while working, effects on work performance, physical impacts, feasibility in the office and so on).

**Table 1:** **Focus group questions**

Questions	
1)	What motivated you to participate in the trial of the sit-stand workstation?
2)	What were your general impressions of using the sit-stand workstation?
Prompts	
a)	How much did you use the sit-stand workstation?
b)	Was there anything about using the sit-stand workstation that you particularly liked?
c)	Was there anything that you particularly disliked?
d)	Any comments about the work surface attached to the workstation?
3)	What types of tasks did you generally do standing up?
Prompts	
a)	Where there times of the day when you stood more?
b)	What made you change from standing to sitting?
c)	Were there any reasons for those decisions?
4)	What types of tasks did you generally do sitting down?
Prompts	
a)	Where there times of the day when you sat down more?
b)	What made you change from sitting to standing?
c)	Were there any reasons for those decisions?
5)	Did anything encourage you to stand up more to complete your work?
Prompts	
a)	Were other people around you using a workstation?
b)	Did certain types of footwear make it easier?
c)	Were you able to stand for longer periods over time?
d)	How long did you tend to stand up for each time?
6)	Was there anything that stopped you from standing more than you did?
Prompts	
a)	Did being in an open plan office make any difference?
b)	Were there any tasks that were not practical while standing?
c)	Was it comfortable to stand and work?
d)	Did you have any injuries or other personal factors?
7)	What types of physical changes did you notice from using the workstation?
Prompts	
a)	Any changes in posture?
b)	Any musculo-skeletal changes?
c)	Any changes in tiredness or energy levels?
d)	Were these related to using the workstation?
8)	What types of changes in your work performance did you notice from using the sit-stand workstation?
Prompts	
a)	Any effect on productivity?
b)	Any effect on ability to concentrate?
9)	Would you continue to use the sit-stand workstation if you could?
Prompt: why/why not?	
10)	In closing, is there anything else you’d like to say about your experience of using the workstation or about your experience of wearing the activity monitors?

### Analysis

We analysed the focus group field notes and transcripts in an iterative process during and after the data collection period to identify the main concepts and themes. The focus group scribe reviewed the field notes following each session and generated a summary of main ideas based on the pre-prepared question guide (a priori themes of interest) and any emergent themes. These focus group summaries were then used as the basis of a framework for coding focus group transcripts (checked and agreed by JYC and MD). Transcripts were scanned for words or phrases that were relevant to these topics and assigned codes using NVIVO Version 10 (QSR International, 2013). All focus group responses are presented by focus group and participant number only, in order to ensure participant anonymity. Due to the small number of participants, who all come from the same workplace, age and gender constituted identifying factors.

## Results

A total of nine focus groups (N = 42 participants) were held from September 2011 to July 2012. Each group had four to five participants, and one person was interviewed individually as they were not able to attend a focus group. Table 2 presents the personal characteristics of the study participants.

**Table 2:** **Participant characteristics of the Stand@Work study**

Characteristic	Mean (SD) or n (%)
N	42
Sex (female)	36 (86%)
Age (years)^a^	38 (11)
Body mass index (kg/m^2^)^b^	
Underweight (<18.5)	5 (13%)
Normal range (18.5 – 24.9)	20 (50%)
Overweight (25.0 – 29.9)	10 (25%)
Obese (≥30.0)	5 (13%)
Highest level of education	
Completed all years of high school	3 (7%)
Trade, technical certificate or diploma	6 (14%)
University	33 (79%)
Working full time	34 (81%)
Office type	
Own office	6 (14%)
Open-plan	36 (86%)

a. Data missing for n = 1.

b. Data missing for n = 2.

### Motivation for participating in the study and trying out a sit-stand workstation

Participants were largely intrinsically motivated to join the trial and these motivations could be grouped into three main themes: 1) curiosity to try something new; 2) interest in potential health benefits; and 3) relevance to the participant’s own and organisation’s work.

Many expressed a curiosity about sit-stand workstations and were interested in trying something new or different at work. The idea of ‘trying before buying’ appealed to some participants, as they did not feel as though they would have to commit to ongoing use if it was not their preference.

*… to give it a test without actually having to commit to it really, long term… the trial aspect was very interesting.* (group 4, participant 1)

Other participants were motivated by potential health benefits that they had heard were associated with sitting less and/or being able to stand while going about their usual daily work in the office. Potential benefits mentioned included musculoskeletal complaints, postural issues, energy levels and cardiovascular health.

*I wanted to know that I wasn’t putting strain on my cardiovascular system and arteries by sitting 8 hours at a time and I just wanted to see if it had a difference to my energy levels and my problems with my back* (group 7, participant 2)

Some participants noted that this research was relevant to their own and their organisation’s work focus. For these participants, being able to contribute to research and workplace policy, as well as experiencing first-hand what it was like to use a sit-stand workstation were motivators for engaging in the trial.

A small number of participants cited extrinsic factors for trialling the sit-stand workstation, such as encouragement from colleagues or managers.

### General impressions of using sit-stand workstations

Participant’s impressions of the sit-stand workstation revolved around three sub-themes 1) ‘surprise and delight’; 2) impact on ability to work; and 3) having choice. Many participants expressed surprise and delight about the sit-stand workstations, and discussed how they used the device more than they thought they would and how they experienced unexpected benefits, such as improved posture and increased alertness. Negative impressions that impacted on user comfort and work ability focused on specific design issues of the sit-stand workstation. A few participants described how they enjoyed having the choice and flexibility to work either sitting or standing positions.

*I just enjoyed the choice, you know, since it’s been gone, you know, I sit there and go “I should stand up and do this task”. Yeah purely for choice and, yep, being able to make that active choice.* (group 5, participant 1)

### Use of sit-stand workstation sitting vs. standing

Participants’ patterns of use of the sit-stand workstation while sitting or standing could be grouped into three sub-categories: 1) task-based routine; 2) time-based routine; and 3) no particular routine.

Some participants took a task-based approach to switching between sitting and standing postures over the course of their workday. For example, they would sit to tackle large word processing tasks, especially those that required using multiple resources and “spreading out” work materials, talk on the phone (for those in the open plan office) and stand to check emails, read documents on the computer screen, or carry out small word processing tasks. It is worth noting there was some variation in the tasks different users preferred to undertake in each position.

*If I need to get stuck into writing something or I need to go through to one of my files, I’ll sit back down again, and then if I’m replying to emails, or you know just working on one document on the screen, then I’ll get back up again.* (group 4, participant 1)

Others described using a time-based routine. One time-based routine involved setting specific durations for sitting and standing (e.g. every 30 or 60 minutes).

*When I saw the clock tick over the hour, I was like, “get up”.* (group 5, participant 1)

*I’d tried to do half hour on and half hour off.* (group 6, participant 1)

*Probably for around about 30 – 40 minute bouts, I suppose, during the day. Probably once every two hours or so.* (group 9, participant 1)

Another time-based routine involved sitting or standing to work based on the time of day. For example some participants indicated they would stand to start the day, after lunch or later in the afternoon, when they felt lower on energy, alertness, and capacity to concentrate.

*I got in and normally tried to stand straight away because I felt good standing up and it was probably best to stand first thing in the morning.* (group 1, participant 3)

*It was better concentrating in the afternoon because you tend to get a bit dozy after lunch, so I think standing after lunch was much better.* (group 3, participant 3)

*It’s a good change for me to be able to stand, and definitely the afternoon is a point where I have a lag. It’s a dead hour after lunch. It’s always hard getting over that hump into the afternoon.* (group 8, participant 4)

Other participants followed no specific routine and simply alternated between sitting and standing postures whenever they felt like it or if they felt tired from being in one posture.

*I didn’t stand for long periods. I stood and sat - I was like a Jack-in-the-box. I was up and down, up and down, rather than standing for a long time and sitting for a while. (*group 2, participant 2)

*I didn’t really assign a pattern to it, I was just like, “oh, I’ve been sitting for a bit, I’ll stand up or I’m getting a bit tired”.* (group 1, participant 3)

A few participants described standing to work as similar to developing a new habit and some viewed being able to stand progressively for longer periods as a personal challenge.

*I used to look at the clock and go “I’ll just do 20 minutes more” and, like, it was always like a little self-competition going.* (group 4, participant 6)

*I think they say it takes 3 or 6 weeks to develop a new habit and this is really kind of like having a new habit.* (group 6, participant 4)

### Barriers to using sit-stand workstation in a standing position

When participants discussed the barriers they faced regarding using the sit-stand workstation in a standing position, they were generally related to either 1) working in the open plan office, or 2) sit-stand workstation design. Some described feeling self-conscious and concerned about disturbing others in the open plan office as inhibitors to standing. This included feeling they were invading their neighbouring colleague’s privacy, revealing confidential communications, and/or disturbing others when talking on the phone.

*I did feel if I was doing something confidential that it was more on show, so I was a little bit wary of that*. (group 3, participant 2)

*When I’m on the phone standing up I feel a little bit conscious because I feel like I’m shouting out across everyone and I’m sort of distracting people next to me.* (group 7, participant 3)

Some participants reported being more distracted when standing up, particularly in the open plan office; yet other participants reported being more focused when standing up to work. There was a view that distractions did or would lessen over time, as more workstation users were in the office and employees adapted to standing as a norm.

*I think if everybody was standing/sitting all the time, nobody would actually care, people would get used to it because if I had people moving all the time and I was standing up, I would probably learn to concentrate more with those distractions.* (group 1, participant 4)

Many participants reported issues with the design of the sit-stand workstation that made it difficult or less comfortable for them to work standing up. These included an unstable work platform when typing, an uncomfortable monitor distance, height restrictions in the standing position for taller users, and the loss of desk space for those users who liked to spread out materials to work.

*I think it kind of depends on your working style, I think if you are somebody who just does nothing with a pen and paper or rarely or very rarely does, it’s perfect because you know you’ve got everything there that you need, but I, I’m a person who often needs to stop typing and starting jotting things down or mapping things out or, you know, with a good old fashion pen and paper, and then you’re like “okay, oh that’s wobbling”*. (group 5, participant 2)

*I found it cut down on my desk space which was a little bit annoying sometimes.* (group 5, participant 1)

Other users also mentioned physical discomfort as a barrier to standing as the ergonomic setup for the sit-stand workstation differed from their usually desk setup. E.g., sore eyes because of the closer monitor distance when upright, wrist discomfort for mouse work

*Sometimes I would be typing as I was standing and going “oh wow I’m getting major pain … in my forearms” and so that was “oh it would be nice if it was already set at your height, perfectly” so you didn’t have to (adjust it every time).* (group 7, participant 2)

#### Facilitators to using the sit-stand workstation in a standing position

Participants discussed a range of factors that encouraged or enabled them to work in a standing position. These can be grouped into three areas 1) a supportive work environment conducive to standing; 2) perceived physical health benefits; and 3) perceived work benefits.

Many participants felt that a supportive work environment helped to normalise standing at work in the open planned space. They said that seeing others standing in the office prompted them to also stand up, and it also created a more sociable work atmosphere and encouraged more personal interaction and communication.

*It does make it a lot more sociable environment… I think it encourages the interaction with going to speak to people as opposed to just always reverting to an email and sitting in your own little silo.* (group 4, participant 2)

*You don’t feel alone, you know, you’re going to stick out like a sore thumb when you are doing work but it’s good when other people do*. (group 8, participant 1)

Some participants described perceived physical improvements, such as reduced back pain and fatigue and increased energy levels, as facilitators to standing more. Others reported improved productivity, alertness, and concentration. There were design aspects that made standing up to work an easy thing to do as well.

*I found my back hurt less as well, ‘cause I’ve got back issues and I found it a lot better, and I was worried about it, you know, maybe it will be weird, but my back has hurt a lot less standing up than sitting.* (group 2, participant 2)

*I felt better at the end of the day. I felt a bit more not as tired at the end of the day.* (group 5, participant 2)

*I was less fidgety I found it really good, cause I could jump up and down and I found I got a lot more done.* (group 2, participant 1)

### Willingness to continue using sit-stand workstations

Participants gave mixed responses when asked whether they would like to continue using the sit-stand workstation after the trial. A majority of participants was in favour of using a sit-stand workstation in the future, but some participants indicated they would prefer a different model workstation.

Some participants did not want to continue to use the sit-stand workstation. It is worth noting however that almost all negative responses related to design issues specific to the sit-stand workstation model that was trialled.

*I don’t want to stand up, but not so much that I would knock it back… I would get used to it and I would build it in to my day but I wouldn’t go looking for it.* (group 6, participant 5)

*Honestly, no and that’s not because I didn’t like standing up as much… it was the desk… I found it quite a challenge with just the way I work.* (group 1, participant 1)

The majority of participants expressed a willingness and desire to keep using a sit-stand workstation. However, few participants indicated that they would like to use sit-stand workstations in the future without further qualifying their response. Nearly all named improvements they would want in the sit-stand workstation and provided an unprompted list of attributes of a sit-stand workstation that they would happily use in the longer term. Suggestions for improvement included having more desk space, less movement in the workstation when in the standing position, and to better adjust the unit for taller participants.

*I would love to have it back, I just think it’s great to have that option of sitting standing and not have to be tied to sitting down all day.* (group 3, participant 2)

*I would actually like one that can be adjusted to be, you know, your height and also can be adjusted as to how far it is from your eyes.* (group 1, participant 2)

Some participants also suggested alternatives to using sit-stand workstations to reduce sitting time in the office, including computer prompts to stand up or having access to a standing ‘hot desk’.

#### Changes in sitting and standing behaviours since the trial

At the end of the discussion, participants had the chance to make general comments about their involvement in the study. Many comments related to increased awareness about the time they spent sitting each day, and their changes in sitting or standing patterns since the sit-stand workstation trial ended and they no longer having had access to the workstation. Examples included choosing to stand more during meetings, or adapting the home environment to sit less. Interestingly, for some, involvement in the study had shifted their view about standing and they were less likely to want or need to sit on public transport during their daily commute.

*I just learnt to tolerate to stand a little longer now. So in a lot of, like, meetings I go to I get up out of my seat and stand.* (group 3, participant 1)

*When I’m at home on my laptop, I take it down to the kitchen bench because it’s high enough for me to stand there and do what I need to do, so I really prefer standing now.* (group 3, participant 1)

*Standing on trains… 70% of my choice and 30% is simply because there are no seats… I’ve sat all day at work so I just would stand but yeah, now I think I like standing more.* (group 6, participant 3)

Some participants noted changes in their own perspective and consciousness about sitting and standing as well as seeing changes in their workplace culture and norms.

*There’s definitely more (standing). I think it’s the awareness of all this happening and I think people think “I can stand for a meeting. It’s okay to stand”… I was actually in an external meeting yesterday with my colleague and she got up and stood up and the facilitator kind of looked at her as if to say “why are you standing up?” … I just thought, you know, it’s quite common in our workplace to do that now, but clearly this woman wasn’t used to that sort of thing happening.* (group 3, participant 2)

## Discussion

This qualitative evaluation of the Stand@Work study presents important formative research that describes office workers’ user experiences and perceptions about the acceptability, and feasibility of a sit-stand workstation modification to their usual office desk. As a relatively new area of intervention evaluation, this study was also an opportunity to assess whether the workplace culture of sitting to undertake desk-based work could be challenged through an environmental modification alone.

The sit-stand workstations were implemented within the study workplace through a collaborative approach. Managers responsible for initiating the trial promoted the study as an opportunity to evaluate how effective and acceptable sit-stand workstations were as a sitting reduction strategy, before committing to a larger roll out, given the lack of evidence to guide decision making. Employees felt they were contributing to both research and organisational health policy, as they were consulted about their views on the workstations while they were given an opportunity to participate in a new area of health promotion that aligned with the organisations purpose. This is reflected in their reasons for participating, which encompassed both individual motives (e.g., trying something new, interest in potential health benefits, flexibility to work standing up) and organisational factors (e.g., relevance to their own and their organisation’s work around workplace wellness and cardiovascular health).

The collaborative engagement of employees in the Stand@Work study is consistent with previous qualitative findings from office workers, that implementing any workplace sitting reduction strategy is both the responsibility of individual employees and organisational management [21], and evidence that greater investment in educating and motivating workers to use sit-stand workstations results in greater uptake [22]. The collaborative approach taken in this study is likely to have contributed to participants’ willingness to try using the sit-stand desk in a standing position. This is consistent with one study where the intervention group comprised sedentary behaviour researchers who were most likely highly supportive of working in a standing posture, educated on the potential health effects of prolonged sitting, and applying ‘sit-less’ strategies in the workplace already with management support [16]. In contrast, another study took a top-down approach where management decided to install a combination of electric powered and manually operated sit-stand desks for all staff in an office refurbishment with little information provided to employers on how to use the new desks, resulting in varying degrees of uptake in desk use while standing up [17].

While the study workplace was a health-related non-government organisation, not all participants described positive user experiences, although most did. Positive feedback about using sit-stand workstations revolved around surprise and delight with the ease of use of the device, having a supportive work environment, and having choice and flexibility in selecting whether and when to sit or stand. Occupational health and safety practitioners have emphasised the importance for workers to have choice over whether they sit or stand to work, and expressed concerns regarding the potential for perceived coercion when implementing any sitting reduction strategy as employees could feel pressured to stand for extended periods, highlighting a tension between optional standing versus compulsory standing [11]. Stand@Work overcame this with clear instructions from the start that participants did not have to stand to work, and that their participation was an evaluation to inform their workplace wellness program and future procurement decisions. Another important issue is that we do not yet have evidence based public health recommendations to guide sit-stand workstation users on how often they should break up sitting time or how long they should stand for in each bout. Current guidelines broadly recommend that adults should reduce the time they spend sedentary or sitting and break up prolonged periods of sitting [23–25]. In our view some caution is needed so that we don’t send a message to stand all day at work either, as this can increase the risk on musculoskeletal problems and varicose veins [26, 27]. Participants in this study were advised to alternate between sitting and standing and build standing time gradually, but then determined their own preferred approach.

Further, participants did not hesitate to give negative feedback about aspects of the study and sit-stand workstations. To illustrate, when asked about their willingness to continue using the sit-stand workstations, the majority of participants responded to the effect of “yes, but…” and provided an unprompted wish list of attributes that they would like to see as part of any new sit-stand workstations that might be purchased in the future. Interestingly, almost all negative feedback related to participant perceptions of design limitations of the model of sit-stand workstation trialled, such as the distance of the computer screen to their eyes, loss of desk space, or platform instability when typing. Thus, it would appear that it was not the act of standing up to work that posed a barrier per se, but rather workstation design, and it is possible that sentiments about future use or considerations about maintenance and sustainability of standing to work may be different should another model of sit-stand workstation, or height adjustable desk be used. In fact, most participants talked about how much they liked having the option to stand to complete desk-based work, even if they did not like the particular device trialled.

We identified several clear patterns of using the workstations in the sitting and standing position in the Stand@Work study. Participants cited task-based, time-based or non-specific routines, with some mentioning they felt they were developing a new habit. Future research could examine whether one approach might be more suitable for certain types of workers or job roles. For instance, some participants reported viewing standing up to work as a personal challenge to develop a new habit; and while this was expressed by only a few participants, it is potentially novel and could be explored in future studies to see if it might be another approach to reducing sitting time.

Conducting phone calls was cited as a barrier to using the workstation standing by a majority of participants in the open plan section of the office, despite being provided with a headset device that made this ergonomically possible. Participants indicated that standing up while on the phone could disturb colleagues in the open plan office and was also an issue when phone calls were confidential. This could present a particular challenge for implementing similar desk modifications in open plan workplaces that have a primary function built around phone calls, such as call centres. Future research on acceptability and feasibility in this setting is recommended.

There were some positive, yet unintended consequences of the trial where participants discussed an increased consciousness about the need to sit less generally and transferred this new awareness into non-work contexts, such as standing up on public transport or when working at home. However, we did not find a quantitative reduction in sitting time in non-occupational domains [20], and this possible transference of reduced sitting into non-work time has not been reported in previous studies. Additionally, we found no indication that standing up to work caused harm to participants and this may be attributable to the emphasis on gradually building up durations of standing time for those who chose to work in a standing position, the ability to easily switch between positions and the relatively short intervention period.

From an employer perspective, one of the potential advantages of this type of environmental modification to reduce sitting is that work time is not interrupted. Usual tasks can still be undertaken in the same location, and from our participant’s feedback, there may also be productivity improvements due to increased alertness, concentration and reduced fatigue, especially later in the day. This needs to be validated by more objective measures, but very few users reported productivity impairments from workstation use. Interestingly, those who were distracted by standing generally felt this lessened over time, as more people were involved in the trial and seeing someone standing up to work was less of a novelty. Since the completion of the trial, the organisation has invested in additional workstations of a newer model that overcomes many of the design limitations reported by users (i.e. taller height limits, wider and more stable keyboard platforms, and sits more stable on an existing desk in the sitting position). Nonetheless, it is too early to make a strong business case for large scale investment in sit-stand workstations, as the health and productivity benefits are yet to be quantified and there is a need for more evidence about longer term use and maintenance of reductions in sitting time reported elsewhere [16–20].

It would also be important to explore lower cost options for reducing sitting because not all workplaces may be able to install or afford sit-stand workstations. Participants reported sitting less in other domains of their day or finding alternatives to break up their sitting time, suggesting that other strategies may be viable as well, whether as complementary or alternative components to using sit-stand workstation and height adjustable desks. Participants also noted the emergence of a ‘sitting less culture’ within the workplace and there are a number of ways this could be encouraged. A menu of sitting reduction options could be designed and provided to workplaces to allow more choice for employers and employees.

### Strengths and limitations

A strength of the study was the collaborative approach to workplace health promotion practice, which facilitates and informs organisational planning in terms of procurement and workplace health promotion. Furthermore, for a qualitative evaluation, the study had a relatively large sample size. A limitation of the study was the use of a convenience sample of participants working in a health-related field who were mostly female, had tertiary education levels, and were of healthy BMI. The generalisability of the results to other office/desk-based workers especially in non-health related workplaces remains unclear. Another limitation of the study was the short term follow-up. Longer term follow up was not possible as participants could only trial the sit-stand workstations for four weeks and we did not have the opportunity to re-assess their behaviours for example at 12 months.

## Conclusion

The use of a sit-stand workstation in this group of desk-based office workers was generally perceived as acceptable and feasible. Most participants were interested in using a sit-stand workstation in the future. Future studies will need to determine the feasibility sit-stand workstations in other populations and settings as well as the feasibility of longer term workstation usage.
